# How Dentists Face the COVID-19 in Mexico: A Nationwide Cross-Sectional Study

**DOI:** 10.3390/ijerph18041750

**Published:** 2021-02-11

**Authors:** Miguel Ángel Casillas Santana, Alan Martínez Zumarán, Nuria Patiño Marín, Brenda Eréndida Castillo Silva, Carolina Sámano Valencia, Marco Felipe Salas Orozco

**Affiliations:** 1 Facultad de Estomatología, Benemérita Universidad Autónoma de Puebla, Puebla C.P. 72000, Mexico; brenda.castillosilva@correo.buap.mx; 2 Department of the Orthodontic Specialty, Facultad de Estomatología, Universidad Autónoma de San Luis Potosí, San Luis Potosí C.P. 78290, Mexico; alanzuma@uaslp.mx; 3 Department of Clinical Research, Facultad de Estomatología, Universidad Autónoma de San Luis Potosí, San Luis Potosí C.P. 78290, Mexico; nuriapaty@uaslp.mx; 4 Dental Materials and Biomaterials Laboratory, Facultad de Estomatología, Benemérita Universidad Autónoma de Puebla, Puebla C.P. 72000, Mexico; carolina.samano@correo.buap.mx

**Keywords:** Mexico, COVID-19, dental practice

## Abstract

**Background**: on 7 January 2020, a new type of coronavirus was isolated, the Severe Acute Respiratory Syndrome Coronavirus2 (SARS-CoV-2), the organism causing the outbreak that has affected the lives of all humans and has modified the rules of coexistence around the world. In Mexico, from 3 January 2020 to 9 January 2021, there have been 1439, 569 confirmed cases of COVID-19 with 131,031 deaths. The World Health Organization reported that Mexico was ranked twelfth, in terms of confirmed cases of COVID-19 by country. **Aim**: the objective of this study was to determine what modifications dentists from the Mexican Republic have made to their dental practice during theCOVID-19 pandemic. **Methods**: the study was conducted based on a questionnaire to evaluate the dentists’ response and knowledge on the modifications in their dental practice to combat the new coronavirus’s cross-transmission. The questionnaire was piloted before it was distributed. The questionnaire was disseminated through the social network Facebook. The questionnaire was distributed to groups of dentists on Facebook, in each of the Mexican Republic states. The survey was carried out during June 2020. **Results and Conclusions:** from the 32 states of the Mexican Republic, 29 participated with at least one respondent. The results of the applied survey suggest that dentists, at least the population of surveyed ones, have proper knowledge of detection methods of patients suspected of COVID-19, preventive measures that must be applied in the dental office to decrease the risk of infection, and the appropriate procedures and solutions for dental office disinfection.

## 1. Introduction

**Figure 1:**
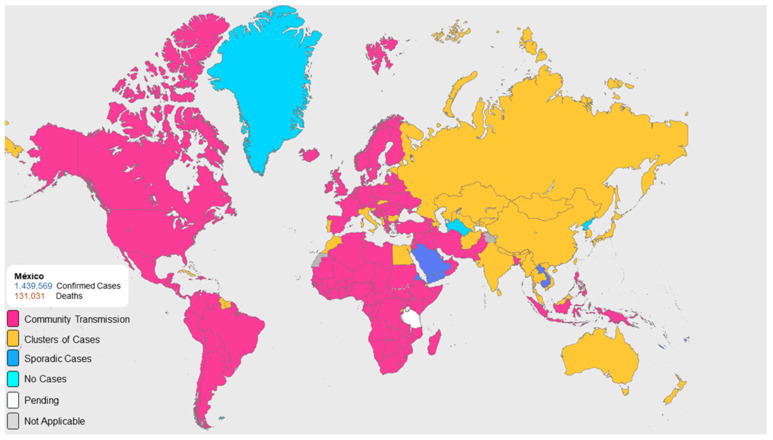
Classification of global transmission as of 17 January 2020, according to the World Health Organization.

On 7 January 2020, a new type of coronavirus was isolated, the Severe Acute Respiratory Syndrome Coronavirus 2 (SARS-CoV-2), the organism causing the outbreak that has affected the lives of all humans and has modified the rules of coexistence around the world [1]. On 11 February 2020, The World Health Organization (WHO) first used the term “COVID-19” to refer to this viral infection, which began in Wuhan, China. This virus structurally comprises a large, single, plus-stranded RNA as its genome, ranging from 60 nm to 140 nm in diameter with spike-like projections on its surface, giving it a crown-like appearance under the electron microscope [2]. The standard clinical features include loss of taste and smell, fever, fatigue, dry cough, myalgia, dyspnea, and other atypical symptoms such as headaches, dizziness, abdominal pain, diarrhea, nausea, and vomiting. The onset of disease may lead to progressive respiratory failure and even death [3,4]. Mexico is in the twelfth place of confirmed cases of COVID-19 globally (Figure 1) [5]. Since the first confirmed case in the country, various efforts have been made to contain the transmission speed of the disease, including widespread confinement and the closure of non-essential businesses. On 16 April 2020, the Mexican Ministry of Health issued recommendations for dental practice; “Prioritize emergencies and postpone ordinary consultations until further notice” [6]. Respiratory viruses can be transmitted from person to person through small or thick droplets of saliva in the air, due to contact with an infected person, or contaminated surface, or through aerosols formed during medical/dental procedures, and due to the characteristics of dental settings; the risk of cross-infection can be very high among dentists and patients [7]. Dentists are a sector of the population with a high potential risk of infection and transmission of COVID-19. The oral conditions that have been reported as rare and that have occurred in confirmed COVID-19 patients, are enlarged lymph nodes in the submandibular region, inflammation in the mouth, dry mouth, ageusia, herpetic lesions, desquamative gingivitis, tongue pain and blisters on the lip mucosa [8]. The prevention measures recommended for dental emergency care in Mexico are the following: give the patient pre-operative antimicrobial mouth-rinse (0.2% povidone iodine and 1% hydrogen peroxide) [9,10], avoid as possible the use of rotary equipment and triple syringe, use of rubber dam, disposable shoe covers, use of disposable gown, surgical goggles or face shield, use of N95 surgical mask and frequently cleaning and disinfecting public contact areas including door handles, chairs and washrooms [4,11]. Additionally, the American Dental Association (ADA) has published recommended preventive measures for dental care; however, dentists may not be aware of recent recommendations [12].

The objective of this study was to determine what modifications dentists from the Mexican Republic have made to their dental practice during the COVID-19 pandemic.

## 2. Materials and Methods

### 2.1. Sample Size Calculation

The required sample size was 387, with an “*n*” of the study universe of 70,000, with a heterogeneity of 50%, a margin of error of 5% and a confidence level of 95%. The sample size was calculated by using the online Netquest calculator.

### 2.2. Questionnaire Desing

After reviewing the relevant literature, international guides, and the Mexican government’s recommendations, the questionnaire was prepared and was initially designed in Spanish. Participants could choose between two options (Yes/No) in questions 1, 9, 10, 12, 13, 14, 15, 17, 19, 20, 21, 22 and 23. The other questions could be answered with more than two options. There was no need to obtain ethics (IRB) approval prior to conducting the survey because the anonymity of the participants was maintained, and they were not coerced to take part in the survey. The questionnaire was pre-tested for validity and reliability in 30 dentists. The reliability of the questionnaire was good, with a Cronbach’s alpha value of 0.84 (*p* < 0.05).

### 2.3. Sample Selection

Once the questionnaire was designed, at least one group of Facebook dentists for each Mexican Republic state was selected to disseminate the survey. The members of each group could voluntarily choose whether or not to participate in the survey. Consecutive non-probabilistic sampling was used. The survey was conducted during June of 2020. The questionnaire was published through a link that led to the Google Forms platform. The questionnaire consisted of 23 questions, which are listed in Table 1.

### 2.4. Statistical Analyses

At the end of the questionnaire application period, a database was obtained in an Excel sheet directly downloaded from the Google Forms platform. When analyzing the database, not all the questions were answered by the total number of dentists surveyed. However, it was decided to include the total number of dentists who answered each question individually to report as much information as possible. The total number of dentists who answered each of the questions is reported in the results section as “total =.” Categorical variables were reported with frequencies and percentages, and the continuous variable (age) was reported with mean and standard deviation. To compare two groups, we used the chi-square (χ^2^) test. Data were analyzed using JMP version 9.0 (SAS Institute, Cary, NC, USA) and Stata version 11.0 (Stata Corp LP, College Station, TX, USA).

## 3. Results

**Figure 2:**
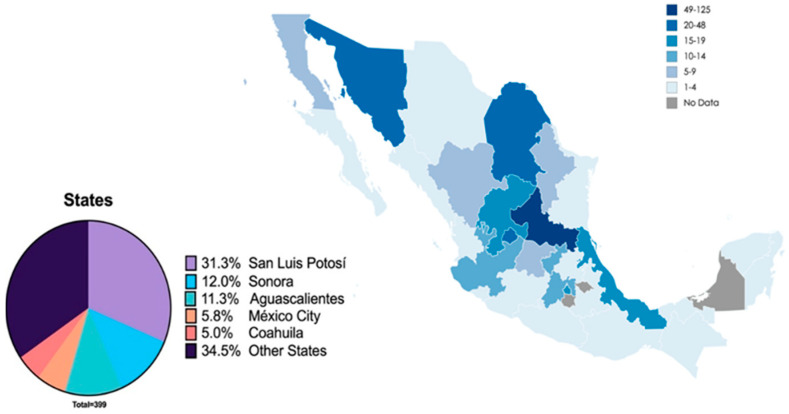
Number and distribution of dentists surveyed by state.

From the 32 states of the Mexican Republic, 29 of them participated with at least one participant. The 5 states with the highest number of respondents were San Luis Potosí (*n* = 125, 31.3%), Sonora (*n* = 48, 12%), Aguascalientes (*n* = 45, 11.3%), Mexico City (*n* = 23, 5.8%) and Coahuila (*n* = 20, 5%) (Figure 2).

**Figure 3:**
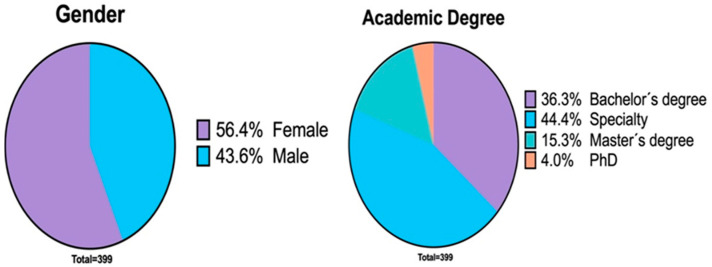
Pie chart showing the distribution of respondents by gender (**left**). Pie chart showing the distribution of respondents by academic degree (**right**).

**Figure 4:**
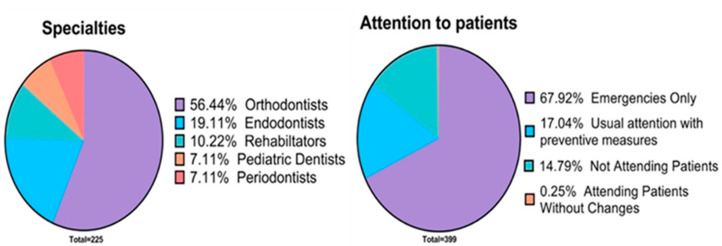
Pie chart showing the specialists who most responded to the survey (**left**). Pie chart showing the type of consultation dentists followed during quarantine (**right**).

A total of 399 dentists answered the survey, from which 225 (56.4%) were women, and 174 (43.6%) were men. Although the total number of participants was 399, not all answered all the questions; therefore, the total number of respondents to each question is indicated in parentheses as “total”. Participants had an average age of 33 ± 11.9 years and a range of 23–77 years. Regarding the degree of studies of the surveyed dentists, most of the dentists who answered the question reported that they had a specialty (total = 339, *n* = 177, 44.4%), followed by a bachelor’s degree (*n* = 145, 36.3%), master’s degree (*n* = 61, 15.3%) and PhD (*n* = 16, 4%) (Figure 3). Concerning the specialties, the dentists who answered the question reported that most of the specialists were orthodontists (total = 225, *n* = 127, 56.4%), followed by endodontists (*n* = 43, 19.1%), rehabilitators (*n* = 23, 10.2%), pediatric dentists (*n* = 16, 7.1%) and periodontists (*n* = 16, 7.1%) (Figure 4). With reference to the master’s and doctorate degrees, the dentists who answered the question reported a great diversity of responses.

In response to the question about what decision the dentist made regarding the management of their daily practice, the majority who answered the question reported that they only attended dental emergencies (*n* = 271, 67%), followed by the option of continuing seeing the same number of patients with the prevention measures (*n* = 68, 17%); and lastly, the option of not treating any patient during the pandemic period (*n* = 59, 14%). Only one of the dentists reported that he had not made any changes in his daily practice.

The preventive measures that the dentists used in their clinical practice are shown in Table 2. The personal protective equipment (PPE) used by the dentists in their clinical practice are described in Table 3. Dentists used a combination of various preventive measures and elements of PPE. Most dentists reported that they know the correct sequence of placement and removal of PPE (total = 337, *n* = 292, 86.6%). Most of the dentists reported that they know the substances for the disinfection of the dental office (total = 339, *n* = 319, 94.1%). Regarding the disinfecting solutions used during the quarantine period, the dentists reported that they used NaOCl (total = 336, *n* = 265, 78.8%), followed by Alcohol at 70% (*n* = 216, 64.3%), glutaraldehyde (*n* = 149, 44.3%) and Lysol (*n* = 8, 2.4%). Lysol is a 50% solution of cresol (3-methyl phenol) in saponified vegetable oil. The rest of the responses covered a wide variety of disinfecting solutions. Only two dentists reported that they were unaware of the recommended disinfectant solutions. Most dentists reported that they know the main symptoms of COVID-19 (total = 336, *n* = 334, 99.4%). Fifty-nine point two percent (total = 336, *n* = 199) of the dentists reported that they were not aware of the possible skin symptoms of COVID-19. Forty-three point three percent (total = 337, *n* = 156) of dentists reported that they do not know the possible oral symptoms of COVID-19, while 53.7% (*n* = 181) reported that they were aware of them.

Regarding the question about the disinfection of the material immediately after the care for a patient, most dentists reported that they disinfect used material immediately after treating the patient (total = 338, *n* = 328, 97%). In reference to the frequency of dental office disinfection, most dentists reported that they disinfect the dental office between each patient (total = 339, *n* = 141, 41.6%), followed by a combination of office disinfection at the beginning and at the end of the day, and between each patient (*n* = 67, 19.8%). Almost all dentists reported that they know where to refer a patient suspected of COVID-19 (total = 338, *n* = 305, 90.2%). The sterilization methods used by dentists during the COVID-19 pandemic are shown in Table 4. Some dentists reported that they use two or more methods of sterilization.

Eighty-three point five percent (total = 339, *n* = 283) of the dentists reported that they have read scientific information regarding COVID-19, while 16.5% (*n* = 56) have not. Ninety-eight point eight percent (total = 338, *n* = 334) of the dentists reported that all the office staff knows and uses the PPE and prevention measures. Eighty-one point four percent (total = 339, *n* = 276) of dentists reported that they do not use any special equipment to decrease or control the generation of aerosols. Finally, 61.8% (total = 338, *n* = 209) of dentists reported a health filter in their dental office.

The variable “knowledge of oral symptoms” was compared to all the other variables present in the study. The differences found between the variables are as follows. The question about oral symptoms of COVID-19 was compared to the question about cutaneous symptoms of COVID-19. According to the results, dentists who identify the oral symptoms of COVID-19 do not identify the cutaneous symptoms of the disease, *p* ≤ 0.0001. In reference to the question about oral symptoms of COVID-19 and whether dentists had sanitation filters at the entrance of the dental office. The results show that dentists who know oral symptoms use sanitation filters at the entrance of their offices, *p* ≤ 0.0032. Comparing the question about oral symptoms of COVID-19 to the question about whether dentists have read scientific information regarding COVID-19. The results indicate that dentists who know the possible oral symptoms of COVID-19, have read scientific information about COVID-19, *p* ≤ 0.0001.

## 4. Discussion

According to the literature review carried out in June 2020 by the authors, for the elaboration of the discussion of this article, eleven articles have been published in which surveys on COVID-19 are applied to dentists. Previously published surveys were applied in various populations of dentists in Italy [13], two in dentists in Jordan [14], in the dentist population of Turkey [15], in the dentist population of India [16], in the dentists from Saudi Arabia [17], in the Netherlands [18], and another one in a population of endodontic specialists in Wuhan [19], in approximately 30 different countries [2], and a multinational survey [1]. Most of these articles focus on investigating the knowledge, attitudes, and procedures of dental practitioners regarding COVID-19.

The dental community response to the questionnaire was meager; this trend was observed in previous studies. The rush to publish this type of information has caused the so-called “hot race to publication”. Therefore, some articles have reported unverified or false information [20].For this reason, it was decided to report each question’s results individually to obtain the highest number of possible answers [21]. The average age of the respondents and the range age reported in this article are similar to those reported in studies in Italy and Jordan [14,21]. Most of the dentists surveyed were women; this also coincides with similar studies [14]. Although there are similar studies in which the majority of the participants were men, for example, in Saudi Arabia (66%) [22].

Generally, it is recommended that dentists only attend emergencies and apply measures to reduce aerosols’ generation [23]. According to the survey results, those Mexican dentists who responded to the survey, reported that they followed the recommendation to only attend dental emergencies (67.9%), while the other percentage chose not to attend any patient during the quarantine (14.8%). Both groups result in 82.71% of all surveyed dentists. These combined percentages are similar to the 71.2% of the dentists in Poland who decided to not treat any patients during the pandemic [24].

Only a small percentage of the Mexican dentists, who responded to the survey, decided to continue their consultation, considering the recommended prevention measures (17%). This percentage is less than the 28.8% of dentists in Poland who decided to continue with their clinical practice [24]. The percentages reported in our study are also similar to those reported in India; where 91.5% of dentists did not provide any type of dental care, while only 8.5% of dentists attended only emergencies [16].

The results show that Mexican dentists who responded to the survey reported that they have tried to follow the general recommendations regarding dental practice. Dentists who continued their dental practice following preventive measures may have been forced to it by various socioeconomic factors [25,26]. Some of the reasons, why the dentists decided to continue their clinical work, may coincide with those reported by Tysiąc-Miśta et al. The three main reasons reported by Tysiąc-Miśta et al. were: not to let the patients suffer pain caused by some dental emergency (59.9%), not to leave the patients unattended (51.6%) and that the economic situation of the dentists forced them to continue with their clinical practice [24].

In relation to the management of dental emergencies, patients should be advised not to go to hospital emergency services since they could come into contact with patients suspected of COVID-19. In the case of dental emergencies in children with special needs, parents should be questioned about the child’s symptoms; this is because children generally do not accurately report their symptoms. Children should attend consultation in the company of only one parent. Upon arrival at the dental office, the temperature of the child and his companion should be taken. They should wait in a waiting room with chairs spaced at least 1.5 m apart and avoid touching as many contact surfaces as possible. At the beginning of the dental consultation, you should be given a 1% hydrogen peroxide mouth rinse. In the case of children with little or null cooperation, the use of handpieces should be avoided, and the performance of dental treatment under general anesthesia should be evaluated [27,28].

The prevention measures most used by the dentists were hand washing, use of PPE, and maintaining a distance of at least 1 m between patients in the waiting room. These prevention measures showed similar percentages in previous studies. For example, the questionnaire applied to dentists in the Lombardy region, in Italy, obtained 78.1% for handwashing and 74.9% for the prevention measure of leaving a space of one meter between patients [21]. The least preventive measures used by the dentists in our study were the chlorination of the water in the dental unit, and the use of techniques to decrease of aerosols’ generation. These measures are vital since the decrease in aerosols is one of the most recommended procedures in dental practice during the COVID-19 pandemic. The chlorination of the water in the dental unit is a measure that should have more diffusion since, together, with the use of mouthwash, it helps to reduce the risk of infection in treatments, in which the generation of aerosols cannot be avoided or when the rubber dam cannot be used [29,30].

Regarding the components of PPE that dentists use, all components have a high percentage of use (70–96%). It is important to note that Mexican dentists who responded to the survey tend to use N95 masks more than dentists in other parts of the world. For example, in the study by Duruk et al. in a population of Turkish dentists, the percentage of use of the N95 mask was only 12% [15]. In the study by Quadri et al. in a population of dentists from Saudi Arabia, only around 40% of the dentists considered the use of the N95 mask as necessary. This percentage is low compared to 70% of Mexican dentists who use the N95 mask. Moreover, this measure somehow compensates for the low use (57.2%) of techniques to decrease the generation of aerosols by Mexican dentists. The use of the N95 mask is widely suggested when the operator is exposed to aerosols [17].

Literature does not recommend using masks made from cotton or gauze since they do not offer any protection neither for the operator nor to the patient. We only recommend the use of surgical masks, which can have different presentations. Surgical masks can be smooth, pleated or rigid, or cup-shaped as the N95 masks. Another type of masks that can be used are respirators. It is crucial to know, in-depth, the correct placement and removal of surgical masks. The mask must cover the nose and mouth; it must be put on before the operator puts on the surgical gloves, and once it is put on, you should avoid touching it with your hands. When removing it, it should be done with bare hands and take it only from the ties that are attached to the ears or the back of the head. If the body of the surgical mask is inadvertently touched, the operator should immediately wash their hands with soap and water for at least 20 s. Masks should be discarded immediately after use in a closed container or bag [31].

The nomenclature for the classification of surgical masks based on the percentage of particles that filter varies in the United States and Europe. The American nomenclature classifies them as N95 and N99. This nomenclature indicates that they filter 95% and 99% of the particles, respectively. The nomenclature of the European classification is as follows: FFP1 masks filter 80% of the particles, FFP2 filters 95% of the particles, and FFP3 filters 99% of the particles. WHO indicates that the same surgical mask can be used for up to a maximum of 4 h. A gown, preferably disposable, with long sleeves and waterproof, should be also worn as part of personal protective equipment. Protective glasses (with side protection) or goggles and masks must be included. A pair of non-sterile gloves should be used since sterile gloves do not provide any extra benefit [32,33].

One of the main prevention measures is taking the patients’ temperature when they arrive at the dental clinic [33]. The Mexican dentists who responded to the survey reported a percentage of use of the infrared thermometer in their clinical practice of 49.9%. The aforementioned percentage is low compared to those reported in other studies. For example, in a worldwide study that included thirty countries, the percentage of use of the infrared thermometer was 81% [2].

Most of the Mexican dentist who responded to the survey reported that they know the correct sequence of placement and removal of the PPE (86.64%). The protective equipment recommended for dental care for patients by the CDC; and its correct placement and removal can be reviewed in detail in the following document https://www.cdc.gov/hai/pdfs/ppe/ppe-sequence.pdf. It is essential that dentists not only know the elements that make up the PPE, but also know its correct placement and removal since this avoids the risk of contagion of COVID-19 towards the operator.

The most widely sterilization method used by the surveyed dentists was the moist heat sterilization method. Likewise, most dentists have verified the effectiveness of the sterilization methods they use. Few dentists (10.62%) use UV light as a sterilization method. The use of UV light has also been proposed for the disinfection of PPE [34]. Disinfection via UV light may be necessary in the case of the N95 surgical masks. Due to the high demand of this type of surgical masks, they can become scarce or have a significantly increase in price. Therefore, the use of UV light for disinfection or sterilization could make them suitable to reuse them [35]. The doses of UV light radiation necessary for disinfection or sterilization of dental material or PPE have already been reported. Dentists who use UV light as a sterilization method or for disinfection can take the reported doses to determine if their UV light kits are effective against COVID-19 [36].

As for the dentist who responded that he had not made any changes in his dental practice. When analyzing his answers to the other questions, we could observe that he uses few preventive measures, like telephone interrogation, the use of N95, gloves and protective glasses. The dentist also reported that he knows the recommended disinfectant solutions. Despite the foregoing, the dentist reported knowing the main symptoms of COVID-19 and performing the disinfection of the dental office only at the beginning and at the end of the day. The dentist also indicated that he does not know the oral or cutaneous symptoms, and that he does not know where to refer suspected patients with COVID-19. Therefore, we can conclude that despite responding that he did not make any changes in his dental practice, the dentist follows the minimum preventive measures, however, these may not be enough to prevent cross infections.

As already mentioned in the results section, dentists who know the oral symptoms of coronavirus have read scientific information about it and tend to apply sanitation filters in their dental offices. The dental community should monitor reports of new symptoms of COVID-19. In addition to the most frequent symptoms such as cough, fever, and general malaise, the symptoms present in patients with moderate symptoms or with uncommon symptoms must be known in detail to add the symptomatology in the questionnaires that are applied to patients before the dental consultation, and thus be able to detect suspected patients with COVID-19 more efficiently. Some unusual symptoms related to COVID-19 infection described in the literature are: itchy or burning eyes, which appeared as the first symptom in a patient in China [37]. Possible oral symptoms have also been reported in suspected or COVID-19 positive patients. Chen et al. published the results of their study, in which a questionnaire applied to COVID-19 positive patients, sought to identify the prevalence of 14 oral symptoms related to COVID-19. However, from the 14 symptoms included in the questionnaire, the authors only present the four oral symptoms with the highest prevalence. The four oral symptoms with the highest prevalence in confirmed COVID-19 patients were: enlargement of the lymph nodes in the submandibular region, inflammation in the mouth, dry mouth, and ageusia. According to this report, the clinical inspection of lymph nodes in the submandibular area before the start of the dental consultation is recommended [8]. Several case reports include herpes lesions, desquamative gingivitis, tongue pain, or blisters on the labial mucosa within the mouth symptoms in confirmed or suspected COVID-19 patients. The oral symptoms of COVID-19 resemble those caused by other types of viral infections, such as the Herpes virus. More studies are needed to confirm that COVID-19 causes these symptoms and to rule out that they are caused by a decrease in the immune system caused by the stress [38].

It is important to note that dentists who knew the oral symptoms were unaware of the coronavirus possible cutaneous symptoms. Therefore, this makes us think that although the dentist seeks information about COVID-19, the search for information is very focused on the effects of COVID-19 on oral health and the search for other types of information is omitted (such as reports of cutaneous symptoms). Knowledge of cutaneous symptoms of COVID-19 can help to complement the questionnaires for the detection of patients suspected of COVID-19. Some of the skin symptoms associated with COVID-19 that have been reported are chilblain or pseudo-chilblain like injury, varicella-like exanthem, maculopapular lesions, and urticarial injury [39].

The results of this study show us interesting information and give us an idea of the management of the pandemic in dental practice in Mexico. This study has some limitations. The first is the low response rate, even though the calculated sample size was achieved. This could have been improved by increasing the time spent on data collection. Another limitation is that the dentists who decided to participate in the survey may be the most likely to follow the prevention measures, therefore, this can be a source of bias. Another limitations is that, it is impossible to verify the veracity of the answers, so the participants were able to choose the options that they thought would be the most appropriate and not the one that would really correspond to their clinical practice. Finally, in 3 states of the Mexican Republic there were no respondents, so there is no information on those 3 states.

## 5. Conclusions

The results of the applied survey suggest that dentists, at least the population of surveyed ones, have a proper knowledge of detection methods for patients suspected of COVID-19, preventive measures that must be applied in the dental office to decrease the risk of infection, and the appropriate methods and solutions for dental office disinfection. We can consider that this level of knowledge is applied to the rest of the population of dentists in the Mexican territory since, by the time of writing this article, the Mexican government has not reported neither any dental health professional to be ill with COVID-19, nor any outbreak of this disease in dental clinics. However, it is crucial to handle the information presented with caution, since the population that answered the call to respond this survey is only a small percentage of the Mexican dentists.

## Figures and Tables

**Table 1:** Questionnaire items.

1. Gender	Male
	Female
2. Age	
3. In which state of the country do you reside?	
4. Level of study:	Bachelor’s degree
	Specialty
	Master’s degree
	PhD
5. What specialty or master’s degree do you have?	
6. What decision did you make regarding your daily consultation during the pandemic?	Not attending patients
	Only attending emergencies
	Continue with the same amount of patients, following the measures of prevention.
	I did not make any changes.
7. If you continue seeing patients, what preventive measures have you implemented in your practice?	Phone triage
	Shoe disinfection mat
	Infrared thermometer
	Handwashing
	Face-to-face questionnaire on patient health
	Space of at least one meter between patients
	Use of personal protective equipment
	Pre-operative rinse with mouthwash
	Chlorination of dental unit water
	Use of techniques that avoid the generation of aerosols
	Use of rubber dam
8. Which of the following personal protection barriers (PPE) do you use in your practice?	N95 face mask
	Face mask other than N95
	Gloves
	Googles or glasses
	Disposable gown
	Disposable surgical cap
	Face shield
9. Do you know the correct sequence of PPE placement and removal?	Yes
	No
10. Do you know the disinfectant solutions for the dental office?	Yes
	No
11. What disinfection solutions do you use in the disinfection of the dental office?	
12. Do you know the main symptoms in patients suspected of COVID-19?	Yes
	No
13. Do you know the skin symptoms that may be present in patients suspected of COVID-19?	Yes
	No
14. Do you know the oral symptoms that can occur in a patient with COVID 19?	Yes
	No
15. Do you carry out any type of disinfection of the material immediately after caring for a patient?	Yes
	No
16. When do you thoroughly disinfect the dental office?	At the beginning of the day
	At the end of the day
	At the end of the morning and evening consultation
	Weekly
	Between each patient
17. If you suspect a patient has COVID 19, do you know where to refer him/her?	Yes
	No
18. What sterilization method do you use in your dental office?	Dry heat
	Humid heat
	Chemical substances
	UV light
19. Have you verified the effectiveness of the sterilization methods you use?	Yes
	No
20. Do dental office staff know and use PPE?	Yes
	No
21. Do you have a sanitation filter at the entrance of your dental clinic or office?	Yes
	No
22. Have you recently read scientific evidence about COVID-19 dental care?	Yes
	No
23. Do you use any special equipment (in addition to the ejectors) for the elimination of the aerosols generated during the dental consultation?	Yes
	No

**Table 2:** Prevention measures used by dentists.

Prevention Measures	Total of Dentists Who Answered the Question	*n* (%)
Phone triage		280 (82.6)
Shoe disinfection mat		280 (82.6)
Infrared thermometer		169 (49.9)
Handwashing		326 (96.2)
Face-to-face questionnaire on patient health		257 (75.8)
Space of at least one meter between patients	339	310 (91.5)
Use of personal protective equipment		329 (97)
Pre-operative rinse with mouthwash		252 (74.3)
Chlorination of dental unit water		134 (39.5)
Use of techniques that avoid the generation of aerosols		194 (57.2)
Use of Rubber dam		218 (64.3)

**Table 3:** Protective barriers used by dentists.

Protective Barriers	Total of Dentists Who Answered the Question	*n* (%)
N95 face mask		238 (70.2)
Face mask other than N95		140 (41.3)
Gloves		331 (97.6)
Googles or glasses	339	309 (91.1)
Disposable gown		310 (91.5)
Disposable surgical cap		280 (82.6)
Face shield		326 (96.2)

**Table 4:** Sterilization method used by dentists.

Sterilization Method	Total of Dentists	*n* (%)
Humid heat sterilization		293 (86.4)
Sterilization by chemical substances	339	167 (49.5)
Dry heat sterilization		78 (23)
UV light		36 (10.6)

## Data Availability

The data presented in this study are available on request from the corresponding author. The data are not publicly available due to privacy.
